# Rescue of endemic states in interconnected networks with adaptive coupling

**DOI:** 10.1038/srep29342

**Published:** 2016-07-06

**Authors:** F. Vazquez, M. Ángeles Serrano, M. San Miguel

**Affiliations:** 1 IFLYSIB, Instituto de Física de Líquidos y Sistemas Biológicos (UNLP-CONICET), 1900 La Plata, Argentina; 2 IFISC, Instituto de Física Interdisciplinar y Sistemas Complejos (CSIC-UIB), E-07122 Palma de Mallorca, Spain; 3 Departament de Física Fonamental, Universitat de Barcelona, Martí i Franquès 1, 08028, Barcelona, Spain; 4 Institució Catalana de Recerca i Estudis Avançats (ICREA), Barcelona 08010, Spain

## Abstract

We study the Susceptible-Infected-Susceptible model of epidemic spreading on two layers of networks interconnected by adaptive links, which are rewired at random to avoid contacts between infected and susceptible nodes at the interlayer. We find that the rewiring reduces the effective connectivity for the transmission of the disease between layers, and may even totally decouple the networks. Weak endemic states, in which the epidemics spreads when the two layers are interconnected but not in each layer separately, show a transition from the endemic to the healthy phase when the rewiring overcomes a threshold value that depends on the infection rate, the strength of the coupling and the mean connectivity of the networks. In the strong endemic scenario, in which the epidemics is able to spread on each separate network –and therefore on the interconnected system– the prevalence in each layer decreases when increasing the rewiring, arriving to single network values only in the limit of infinitely fast rewiring. We also find that rewiring amplifies finite-size effects, preventing the disease transmission between finite networks, as there is a non zero probability that the epidemics stays confined in only one network during its lifetime.

Most biological and sociotechnological systems in nature are not isolated, but they are formed by a set of complex networks which interact following intricate patterns. These systems are conveniently represented in terms of a multilayer system of interconnected network layers^1^,^2^, in which each layer describes a different type of population. A different multilayer context is that of multiplex networks, in which the same nodes exist in different network layers. The function of multilayered structures, like sustaining diffusion processes, is characterized by emerging features which are not observed in single-layered systems^3^,^4^. In particular, epidemics propagating on two interconnected networks show a direct dependence on the patterns of interconnection between the layers. A previous work on the Susceptible-Infected-Susceptible (SIS) model^5^ showed that multilayered interconnected systems can support an endemic state even if each isolated network is not able to do so, and that internetwork correlations boost the propagation on the two-network system. In a related work^6^, the authors found a mixed phase in the Susceptible-Infected-Recovered (SIR) dynamics –besides the endemic and the healthy phase observed in isolated networks–, in which the epidemics spreads in one network only. The SIS and SIR dynamics have also been explored in multiplexes^7^,^8^,^9^,^10^,^11^ and in interconnected networks where the disease involves different competing species^12^,^13^. Moreover, general spreading processes has recently been studied in multilayer networks^14^.

These and many other works on epidemic spreading on multilayered systems considered static links inside and between layers, since contacts between individuals are assumed to be constant over time. However, it is known that the frequency and duration of face-to-face contacts are quite heterogeneous, and follow non-trivial patterns^15^. Past studies in single networks incorporated different mechanisms of time varying topology and network adaptation^16^. A prominent example of time dependent networks are coevolving networks, in which the state of each node is influenced by, and simultaneously shapes, the local network structure^17^,^18^,^19^,^20^,^21^. This coupling of the dynamics *on* the network with the dynamics *of* the network leads to a rich variety of different long term behaviors of the system. Simple contact processes like the SIS model^22^, the voter model^23^,^24^ or the Axelrod model^25^ on coevolving networks give rise to new phenomena, not seen in static networks, such as self-organization towards critical behavior, the formation of complex topologies and network fragmentation transitions. Coevolution in multilayer networks is usually studied in two extreme limits, depending on the relative difference between the time scales associated to the evolution of intralayer and interlayer links. The limiting case of adaptive intralayer links with fixed interlayer links has recently been considered^26^,^27^. In this article we address the opposite limit of fixed intralayer links and adaptive interlayer links in a bilayer system, representing two interconnected networks. A motivation for this general situation of interconnected networks is the case of the power-grid and Internet networks^28^, where links between them are reconnected to replace failing connections. In the context of epidemic spreading, the situation we consider could model the case of sexual disease transmission^5^. Homosexual and heterosexual layers are connected by bisexual relations that are assumed to be more volatile than intralayer relations, because of their sporadic character. Another possible application could be in the study of epidemic spreading in two geographically distant populations^26^ (the two layers), with interlayer links being commuters between them, which are more volatile relations than those that take place within each geographical population.

In this article we study the SIS epidemic spreading on two interconnected networks with dynamic connections between layers. Links connecting susceptible and infected individuals at the interlayer are rewired and reconnected to a random pair of susceptible individuals. In this way, the dynamics of interlayer links is coupled to the dynamical evolution of the states of the nodes. We find that the network adaptation process associated with the rewiring of interlayer links can dramatically reduce the spreading of the disease within and between layers. We consider two different scenarios. In weak endemic states, when the epidemics spreads only if the two layers are interconnected, we find a critical rewiring rate beyond which the system remains in the healthy phase. For strong endemic states, in which the epidemics is able to spread on each separate network, we find that rewiring is specially effective in preventing spreading on finite systems. As we shall see, link adaptation amplifies finite-size effects, leading to a finite probability that the epidemics stays confined in only one layer during its lifetime.

## Results

### Model and mathematical framework

The topology of the system consists on two Erdös-Renyi networks (or layers) A and B, interconnected by adaptive links. Each network has the same number of nodes *N*_*A*_ = *N*_*B*_ = *N* and average degrees 〈*k*_*A*_〉 = 〈*k*_*B*_〉 = 〈*k*〉. The number of links between layers (interlinks) *q*〈*k*〉*N*/2 is proportional to the number of links inside each layer (intralinks) 〈*k*〉*N*/2, where the factor *q* ≥ 0 is a measure of the intensity of the coupling between the networks, so that for *q* = 0 the networks are totally decoupled. As a result, nodes in each network have an average number 〈*k*〉 of intranetwork connections and 〈*k*_*AB*_〉 = *q*〈*k*〉/2 of internetwork connections.

An SIS epidemics spreads on top of the coupled system. Nodes can be in one of two possible states, either susceptible (state *S*) or infected (state *I*). Each infected node transmits the disease to its susceptible neighbors at rate *λ* (*λ*_*A*_ and *λ*_*B*_ inside each layer, and *λ*_*AB*_ (*λ*_*BA*_) from nodes in layer A (B) to nodes in layer B (A)), and recovers becoming susceptible again at rate *μ*, that without loss of generality we take as *μ* = 1. Intralayer links in A and B are fixed in time, but interlayer connections between A and B are allowed to adapt over time. That is, every interconnection between an infected node in network A (B) and a susceptible node in B (A) is randomly rewired at rate *ω*. To implement the rewiring, the selected Infected-Susceptible (*IS*) interlink is removed and a new Susceptible-Susceptible (*SS*) interlink is created in its place, in which both susceptible nodes are chosen at random in the different layers. The number of links remains unchanged in a rewiring event, thus the total number of links in the system is conserved at all times. The qualitative behavior of the system is expected to be the same if the rewiring is made from an *IS* pair to another randomly chosen *SS* or *IS* pair at the interlayer.

We now develop a mathematical approach that allows to study the dynamics of the system in terms of the time evolution of the global densities of nodes and links in the different states. The approach is based on a mean-field description at the level of pairs of nodes, and assumes that the system is infinitely large. We denote by *I*_*A*_ and *S*_*A*_ the densities of infected and susceptible nodes in network A, respectively, and by *I*_*A*_*S*_*A*_, *I*_*A*_*I*_*A*_ and *S*_*A*_*S*_*A*_ the densities per node of intranetwork links in A connecting an infected and a susceptible node, two infected nodes and two susceptible nodes, respectively. An analogous notation is used for network B. For interconnections, we use the notation *I*_*A*_*S*_*B*_ (*S*_*A*_*I*_*B*_) to represent the density per node of internetwork links between an infected node in A (B) and susceptible node in B (A), while *I*_*A*_*I*_*B*_ and *S*_*A*_*S*_*B*_ stand for the densities of interlinks between two infected nodes and two susceptible nodes, respectively. We note that these quantities are symmetric respect to the subindices A and B.

Given that the number of nodes in each layer is conserved, as well as the number of intra and interlinks, the following conservation relations hold at any time:


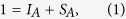



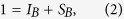



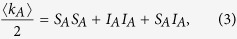



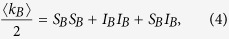



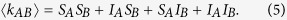


In order to obtain a closed system of equations for the evolution of the densities we make use of the *pair approximation* (see section I of the Supplementary Information), which assumes that the links of different types are homogeneously distributed over the networks, and thus the densities of triplets can be written in terms of the densities of pairs. We obtain


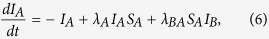



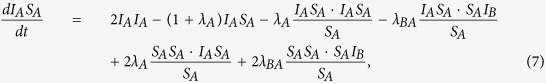



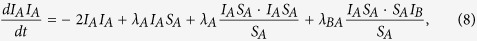



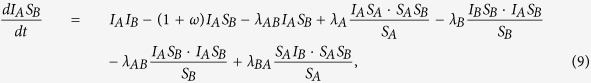



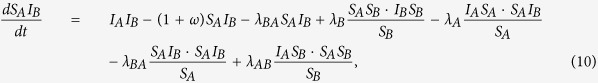



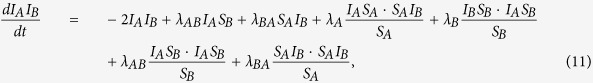


plus the rate equations for *I*_*B*_, *I*_*B*_*S*_*B*_ and *I*_*B*_*I*_*B*_, which have the same form as the equations for *I*_*A*_, *I*_*A*_*S*_*A*_ and *I*_*A*_*I*_*A*_, respectively, after interchanging sub-indices *A* and *B*.

The pair approximation works reasonably well for networks with homogeneous degree distributions and low degree correlations, such as Erdös-Rényi (ER) or degree-regular random graphs, but may not be accurate enough for non-homogeneous or correlated topologies, like scale-free networks. Even for ER networks, quantitative disagreements between the simulations and the analytical solution may arise in some regimes due to dynamic correlations not captured by the approximation, as it happens when the system is close to the healthy absorbing state^29^.

The set of non-linear coupled ordinary differential Eqs (6)–(11) [Disp-formula eq7][Disp-formula eq8] [Disp-formula eq9][Disp-formula eq10] [Disp-formula eq11] together with the conservation laws Eqs (1)–(5) form a closed set of equations for the system’s dynamics. This mathematical framework is suitable to study macroscopic quantities, such as the prevalence of the epidemics (stationary density of infected nodes *I*_*A*_ and *I*_*B*_) in each layer. We are particularly interested in the effects of the rewiring on the prevalence, thus we focus on the dependence of *I*_*A*_ and *I*_*B*_ on the rewiring rate *ω*. We shall see that the behavior of the coupled system at the stationary state differs qualitatively depending on whether the global endemic state is *weak* or *strong*, with strong endemic states having the capacity to propagate on each network separately, while weak endemic states are unable to do so^5^. We recall that strong endemic states are supported by infectivity levels above the critical value *λ*_*c*_ in each single layer, while weak endemic states appear whenever the infectivity is below the single epidemic threshold *λ*_*c*_, but above the epidemic threshold *λ*_*c*,*q*_ of the interconnected system, which depends on the coupling *q*. In single random uncorrelated networks the critical threshold is typically given by the expression *λ*_*c*_ = 〈*k*〉/〈*k*^2^〉^30^, and it can be better approximated by *λ*_*c*_ = 〈*k*〉/〈*k*(*k* − 1)〉^5^, which corrects in part the effect of dynamical correlations. In ER networks 〈*k*^2^〉 = 〈*k*〉^2^ + 〈*k*〉, and thus the single-layer threshold is reduced to the simple expression *λ*_*c*_ = 1/〈*k*〉^31^.

### Weak endemic states

As we mentioned above, the existence of endemic states in the coupled system of two layers is possible even when infection rates are below the critical rate *λ*_*c*_ of a single layer, as was shown in ref. 5. It turns out that the infection in each layer is reinforced by its partner layer through the interconnections, and thus the entire system lowers its epidemic threshold to a new value *λ*_*c*,*q*_ < *λ*_*c*_, associated to the interconnectivity *q*. Therefore, endemic sates are also observed for infection rates in the region *λ*_*c*,*q*_ ≤ *λ* ≤ *λ*_*c*_. In this section we focus on the *λ* < *λ*_*c*_ case, and study the behavior of the system when interconnections are rewired at a given rate *ω*.

**Figure 1:**
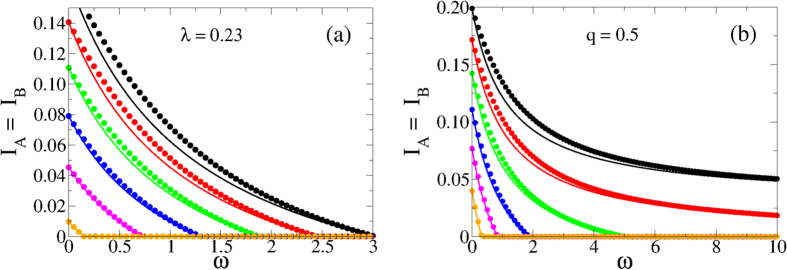
Disease prevalence *I*_*A*_ = *I*_*B*_ in a symmetric two-network system vs the rewiring rate *ω*. Each network has average degree 〈*k*〉 = 4. Circles are the results from the numerical integration of Eqs (6)–(11) [Disp-formula eq7][Disp-formula eq8] [Disp-formula eq9][Disp-formula eq10] [Disp-formula eq11], while lines are the analytical approximation Eq. (20). (**a**) For infection rate *λ* = 0.23 and values of the coupling *q* = 0.7, 0.6, 0.5, 0.4, 0.3 and 0.2 (from top to bottom). (**b**) For *q* = 0.5 and values of the infection rate *λ* = 0.26, 0.25, 0.24, 0.23, 0.22 and 0.21 (top to bottom).

In Fig. 1 we show the prevalence *I*_*A*_ = *I*_*B*_ in networks A and B vs *ω*, as predicted by Eqs (6)–(11) [Disp-formula eq7][Disp-formula eq8] [Disp-formula eq9][Disp-formula eq10] [Disp-formula eq11], for a symmetric system with 〈*k*〉 = 4 and infection rates *λ*_*A*_ = *λ*_*B*_ = *λ*_*AB*_ = *λ*_*BA*_ = *λ*. We observe that the prevalence decreases as the rewiring increases, and vanishes at a finite value 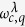 when *λ* < *λ*_*c*_ = 1/〈*k*〉 = 0.25. That is, there is a continuous transition from an endemic to a healthy phase when the rewiring overcomes a critical value 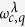, which depends on the coupling *q*, the infection rate *λ* and the average connectivity 〈*k*〉. In order to derive an analytical expression for 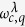 we use the system of Eqs (6)–(11) [Disp-formula eq7][Disp-formula eq8] [Disp-formula eq9][Disp-formula eq10] [Disp-formula eq11] and study the stability of the healthy phase under a small perturbation, which consists on infecting a small fraction of nodes in both layers. We consider the symmetric case scenario in which both perturbations are the same, so that initially *I*_*A*_ = *I*_*B*_ ≪ 1. Because of the symmetries of the system and initial conditions, the prevalence in both networks must be the same, thus *I*_*B*_*S*_*B*_ = *I*_*A*_*S*_*A*_, *I*_*B*_*I*_*B*_ = *I*_*A*_*I*_*A*_ and *S*_*A*_*I*_*B*_ = *I*_*A*_*S*_*B*_. This reduces Eqs (6)–(11) [Disp-formula eq7][Disp-formula eq8] [Disp-formula eq9][Disp-formula eq10] [Disp-formula eq11] to a system of five equations with five unknown densities: *I*_*A*_, *I*_*A*_*S*_*A*_, *I*_*A*_*I*_*A*_, *I*_*A*_*S*_*B*_ and *I*_*A*_*I*_*B*_. Given that these densities depend on the number of infected nodes *I*_*A*_ ≪ 1, they are also very small initially. Then, we linearize the equations around the fixed point *S*_*A*_ = *S*_*B*_ = 1, *S*_*A*_*S*_*A*_ = *S*_*B*_*S*_*B*_ = 〈*k*〉/2, *S*_*A*_*S*_*B*_ = 〈*k*_*AB*_〉 = *q*〈*k*〉/2, by neglecting terms of order 2 or higher in the five densities. We obtain a reduced system that can be written in matrix representation as


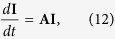


where


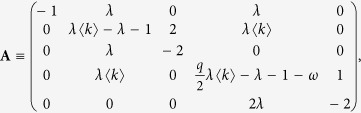


and


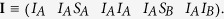


At the critical point, the determinant of **A** must be zero, from where


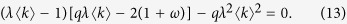


Finally, solving for *ω* we obtain the critical rewiring rate


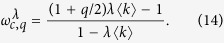


**Figure 2:**
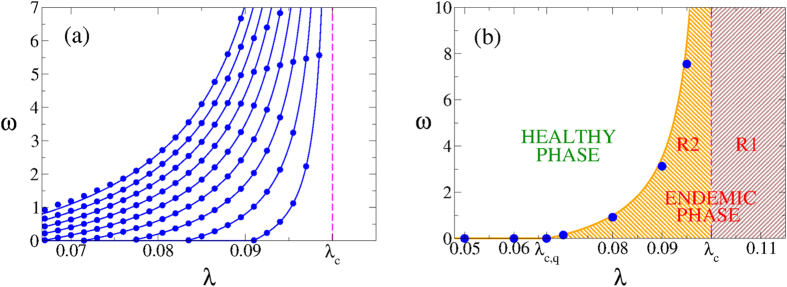
Critical rewiring rate 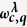 vs infectivity *λ* for a symmetric two-network system. Networks have average degree 〈*k*〉 = 10. (**a**) Results from Eqs (6)–(11) [Disp-formula eq7][Disp-formula eq8] [Disp-formula eq9][Disp-formula eq10] [Disp-formula eq11] (circles) and from Eq. (14) (solid lines), for values of the coupling *q* = 0.2, 0.4, 0.6, 0.8, 1.0, 1.2, 1.4, 1.6 and 1.8 (from right to left). (**b**) Healthy-endemic phase diagram for two coupled ER networks of *N* = 10^6^ nodes each, average degree 〈*k*〉 = 10, and coupling *q* = 1.0. One-layer and two-layer critical infection rates are *λ*_*c*_ = 0.1 and *λ*_*c*,*q*_ = 0.067, respectively. Filled circles are results from simulations, while the solid line is the analytical solution Eq. (14) of the system of Eqs (6)–(11) [Disp-formula eq7][Disp-formula eq8] [Disp-formula eq9][Disp-formula eq10] [Disp-formula eq11].

In Fig. 2(a) we plot 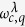 vs the infectivity *λ* for various values of *q* and 〈*k*〉 = 10, obtained from Eq. (14) (solid lines). We observe that the agreement with the integration of Eqs (6)–(11) [Disp-formula eq7][Disp-formula eq8] [Disp-formula eq9][Disp-formula eq10] [Disp-formula eq11] (circles) is perfect. As we can see, 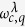 is larger for larger values of *q* and *λ*, and diverges as *λ* approaches *λ*_*c*_ = 1/〈*k*〉 = 0.1. This divergence means that for strong endemic states (*λ* > 0.1) there is no finite rewiring able to stop the spreading. In the next section, we explore this phenomenon in more detail.

In order to compare analytical results with Monte Carlo (MC) simulations of the dynamics, we run simulations on two coupled ER networks with *N* = 10^6^ nodes and mean degree 〈*k*〉 = 10 each, and coupling *q* = 1.0. Results are shown in Fig. 2(b). To estimate the critical rewiring from MC simulations we performed spreading experiments (see section “Methods” for details). We observe a good agreement between simulations (circles) and the prediction from Eqs (6)–(11) [Disp-formula eq7][Disp-formula eq8] [Disp-formula eq9][Disp-formula eq10] [Disp-formula eq11] (solid line).

A more detailed analysis of the phase diagram of Fig. 2(b) can be obtained by doing a stability analysis of the system of equations (12). All real parts of the eigenvalues of the Jacobian matrix **A** must be strictly negative for the healthy state solution **I** = **0** to be stable. Then, a sufficient condition for the instability is the existence of a positive real part of one of the five eigenvalues. This happens in two regions *R*_1_ and *R*_2_ of the *ω* − *λ* phase space that fulfill the conditions


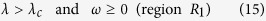


or


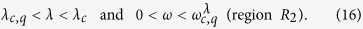


Regions *R*_1_ and *R*_2_ make up the endemic phase [see Fig. 2(b)]. According to Eq. (15), when *λ* is above the epidemic threshold of the single network *λ*_*c*_ the epidemics propagates on the coupled system, independently of the rewiring value. This is in agreement with the fact that in region *R*_1_ the critical rewiring 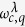 from Eq. (14) is negative for any value of the parameter’s topology. Since *ω* is meaningful only for positive values there is no critical rewiring able to stop the propagation of the epidemics. At the single layer threshold *λ*_*c*_ the critical rate 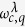 diverges, and it is above the region *R*_2_ that for finite values of 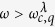 the two-network system becomes effectively decoupled, and the epidemics cannot propagate. Thus, a high enough rewiring rate can induce an effective rescue of weak endemic states.

From the eigenvalue Eq. (13), we can also obtain the critical infection rate


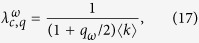


where


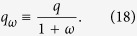


**Figure 3:**
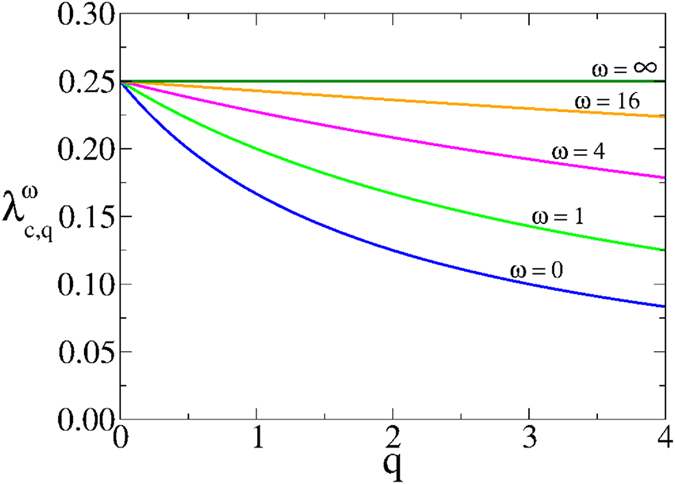
Critical infection rate 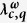 of a system of two interconnected networks vs the coupling factor *q*. Each network has average degree 〈*k*〉 = 4. Solid lines represent the expression Eq. (17) for different values of the rewiring rate *ω* = ∞, 16, 4, 1 and 0 (from top to bottom).

Equation (17) corresponds to the infection threshold for the general case of two networks with coupling *q* and rewiring *ω*. In the absence of coupling *q* = 0, Eq. (17) reduces to the expression for the single network threshold *λ*_*c*_ = 1/〈*k*〉. In the case of static coupling *q* > 0 and *ω* = 0, the threshold is


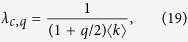


which corresponds to the critical infection rate of a single ER network with average degree (1 + *q*/2)〈*k*〉. This is so because the two-layer system can be interpreted as one layer with a total mean degree composed by intralink term 〈*k*〉 and an interlink term 〈*k*_*AB*_〉 = *q*〈*k*〉/2. Finally, in the dynamic coupling case *q* > 0 and *ω* > 0, the factor *q*_*ω*_ plays the role of an *effective coupling* when the rewiring is present, given that Eq. (17) has the same structural form as Eq. (19) in a static two-layer network with coupling *q*_*ω*_. Figure 3 shows the behavior of 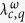 from Eq. (17). The critical infection rate decreases with *q* and grows with *ω*, from *λ*_*c*,*q*_ (for *ω* = 0) to *λ*_*c*_ (for *ω* → ∞).

As we see from Eq. (18), the effect of rewiring is to reduce the coupling between the two layers for the transmission of the disease, even though the “structural coupling” *q* is not affected by *ω*. Therefore, we can think the two-layer system with *dynamic* interconnections rewired at rate *ω* as a system with *static* interconnections but with an effective reduced coupling *q*_*ω*_. We need to mention that this conclusion is strictly valid only at the transition point, as it follows from Eq. (18). An important consequence is that *q*_*ω*_ → 0 in the infinitely fast rewiring limit *ω* → ∞, and thus the networks become totally decoupled in terms of the disease transmission between them.

In section II of the Supplementary Information we take advantage of the mapping between dynamic and static interconnections and derive an approximate solution of Eqs (6)–(11) [Disp-formula eq7][Disp-formula eq8] [Disp-formula eq9][Disp-formula eq10] [Disp-formula eq11] for a symmetric system, which reads


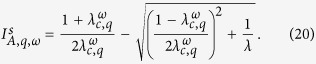


In Fig. 1 we observe that the approximate solutions from Eq. (20) (solid curves) are in perfect agreement with the numerical integration of Eqs (6)–(11) [Disp-formula eq7][Disp-formula eq8] [Disp-formula eq9][Disp-formula eq10] [Disp-formula eq11] (circles) only at 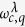 and *ω* = 0, but deviations arise between these values because the mapping is not exact for all values of *ω*.

### Strong endemic states

We explore in this section how the rewiring affects the epidemics when infection rates are above the single layer threshold *λ*_*c*_. In this case, the endemic state can be sustained in each network separately, but we shall see that when the layers are interconnected the prevalence depends on the coupling *q* and the rewiring rate *ω*. We first make the analysis using the rate equations, which corresponds to the behavior on infinite large systems (thermodynamic limit). Then, we study the case of finite systems, where new phenomenology appears due to the extinction of the epidemics by finite size fluctuations.

#### Thermodynamic limit

**Figure 4:**
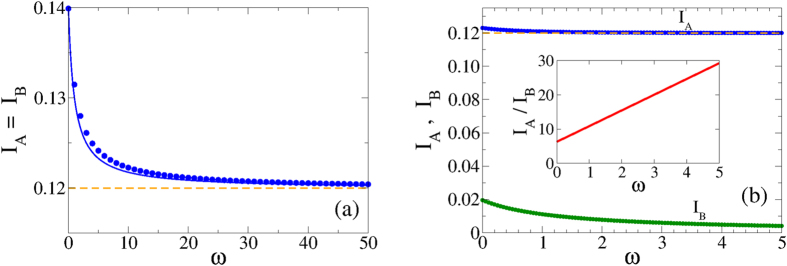
Prevalence *I*_*A*_ and *I*_*B*_ in networks A and B as a function of the rewiring rate *ω*. Average degree and coupling are 〈*k*〉 = 10 and *q* = 0.5, respectively. (**a**) The infectivities are *λ*_*A*_ = *λ*_*B*_ = *λ*_*AB*_ = *λ*_*BA*_ = *λ* = 0.115 above the single layer critical rate *λ*_*c*_ = 0.1. Circles correspond to the integration of Eqs (6)–(11) [Disp-formula eq7][Disp-formula eq8] [Disp-formula eq9][Disp-formula eq10] [Disp-formula eq11]. The dashed line represents the prevalence level 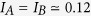 from Eq. (21), while the solid curve is the analytical approximation Eq. (20). (**b**) The infectivities are *λ*_*A*_ = *λ*_*AB*_ = 0.115 and *λ*_*B*_ = *λ*_*BA*_ = 0.085, above and below *λ*_*c*_, respectively. The prevalence in layer B (lower curve) is zero when is isolated, while the prevalence in layer A (upper curve) approaches its single layer value 0.12. Inset: ratio between prevalence in networks A and B as a function of *ω*.

In Fig. 4 we show the prevalence levels *I*_*A*_ and *I*_*B*_ as a function of the rewiring rate *ω*, obtained from the numerical integration of equations (6)–(11), using 〈*k*〉 = 10 and 〈*k*_*AB*_〉 = 0.25. Figure 4(a) corresponds to infection rates *λ*_*A*_ = *λ*_*B*_ = *λ*_*AB*_ = *λ*_*BA*_ = *λ* = 0.115 above the epidemic threshold *λ*_*c*_ = 1/〈*k*〉 = 0.1, thus each separate network is in the endemic state. As expected, *I*_*A*_ and *I*_*B*_ reach the same stationary value. We observe that the prevalence decreases monotonically as *ω* increases, and approaches the prevalence level 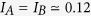 in isolated networks when *ω* becomes very large (horizontal dashed line). This asymptotic value corresponds to the analytical expression


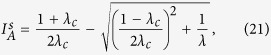


derived in section II of the Supplementary Information, for the prevalence in single networks. This behavior is reminiscent of what we found in the last section, that is, both networks become effectively decoupled as *ω* goes to infinity, leading to stationary values *I*_*A*_ and *I*_*B*_ corresponding to single isolated networks. This happens because when the rewiring is sufficiently large the number of *IS* links at the interface is reduced to zero. Then, spreading is not possible between networks, given that the disease is transmitted through infected nodes and, therefore, layers are completely decoupled for transmission. The absence of *IS* interlinks in the *ω* → ∞ limit can be derived from Eqs (6)–(11) [Disp-formula eq7][Disp-formula eq8] [Disp-formula eq9][Disp-formula eq10] [Disp-formula eq11]. In this limit, the dominant term on the right hand side of Eq. (9) is −*ωI*_*A*_*S*_*B*_, which gives the exponential decay *I*_*A*_*S*_*B*_(*t*) ~ *e*^−*ωt*^. That is, the stationary density of *I*_*A*_*S*_*B*_ pairs is zero. Following the same argument we obtain from Eq. (10) that the stationary density of *S*_*A*_*I*_*B*_ pairs is zero as well. Then, using these two stationary values in Eq. (11) we get that *I*_*A*_*I*_*B*_ is also zero. In other words, the system quickly evolves to a stationary state characterized by the absence of interlinks connecting infected nodes.

As it was shown in ref. 5, it is not possible to find a mixed phase in the coupled static system, that is, a phase that consists on an endemic state in one layer and a healthy state in the other layer. This is so because when the disease is able to propagate on one layer, then it always propagates on the entire system. This phenomenon is also observed even if the interlinks are rewired, as we see in Fig. 4(b), where we show results from Eqs (6)–(11) [Disp-formula eq7][Disp-formula eq8] [Disp-formula eq9][Disp-formula eq10] [Disp-formula eq11] with infection rates *λ*_*A*_ = *λ*_*AB*_ = 0.115 > *λ*_*c*_ = 0.1 and *λ*_*B*_ = *λ*_*BA*_ = 0.085 < *λ*_*c*_ = 0.1. Under these asymmetric conditions, the disease is able to spread in layer A but it dies off in layer B, when the networks are disconnected. Again, finite rewiring rates reduce the prevalence in each layer but cannot induce a complete breaking of the coupling, which only happens in the *ω* → ∞ limit.

#### Finite size effects

Up to know we studied the prevalence of the disease in both networks under different scenarios, and how this prevalence is affected by the rewiring of internetwork links, as compared to the case of static interconnections. The analysis was done by means of a system of equations for the densities of nodes and pairs, which is a good mean-field description of the disease dynamics in infinite large networks, where finite size fluctuations are neglected. This allows to study various phenomena that are independent on the system size for large enough networks, such as the transition between the endemic and the healthy phase when *λ* and *ω* are varied. These equations predict that the disease in the endemic phase last for infinite long times. However, in finite networks, fluctuations in the density of infected nodes in a given realization eventually drive the system to an absorbing configuration, where all nodes are susceptible. This brings new interesting phenomena that are intrinsic of finite systems, and that we explore in this section.

**Figure 5:**
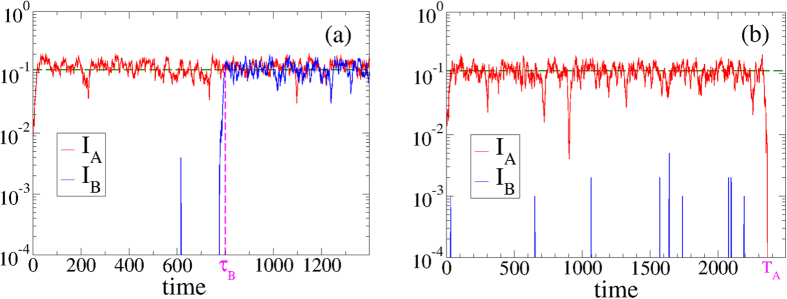
Time evolution of the density of infected nodes *I*_*A*_ and *I*_*B*_ in two coupled ER networks A and B, in two distinct realizations of the dynamics (**a,b**). Networks have average degree 〈*k*〉 = 10 and *N* = 10^3^ nodes each, coupling *q* = 0.005, interlink rewiring *ω* = 50, and *λ*_*A*_ = *λ*_*AB*_ = *λ*_*B*_ = *λ*_*BA*_ = *λ* = 0.115 > *λ*_*c*_ = 0.1. In realization (**a**) there is an outbreak in both layers, while in realization (**b**) the outbreak occurs in layer A only.

We are interested in studying whether the rewiring is able to stop the spreading of the disease from network A to network B, when fluctuations are taken into account. As we showed in the last section for infinitely large systems, when either *λ*_*A*_ > *λ*_*c*_ or *λ*_*B*_ > *λ*_*c*_ the disease spreads over both networks, independently on the initial condition and the rewiring rate. In other words, if the disease spreads on A then it spreads on B with probability equal to unity. Only in the *ω* → ∞ limit internetwork spreading is stopped, because networks are effectively disconnected. However, we shall see that this scenario is no longer valid in finite networks, where in a single realization of the dynamics with finite *ω* (including *ω* = 0), the spreading can take place in one network only. Indeed, Fig. 5(a,b) show two distinct realizations with the same parameters and initial condition, but with very different outcomes. Initially, only one random node in network A and its neighbors are infected. We investigate whether this initial small focus of infection in network A is able to spread on A and invade network B. In Fig. 5(a), the disease in network A quickly spreads until it reaches a stationary state in which *I*_*A*_ fluctuates around a given value 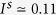 (horizontal dashed line). We refer to this state where a large fraction of the population gets infected as an *outbreak*. In network B the outbreak happens at a much longer time 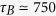, but eventually the disease spreads on both networks, as it happens in infinite systems. However, in Fig. 5(b) there is also a quick outbreak in network A but the outbreak in network B never happens, because *I*_*A*_ becomes zero by a large fluctuation at time *T*_*A*_, driving the entire system to a halt. That is, the epidemics on A goes extinct before the disease can spread on network B. Below we present results corresponding to the statistics associated to the outbreaks.

**Figure 6:**
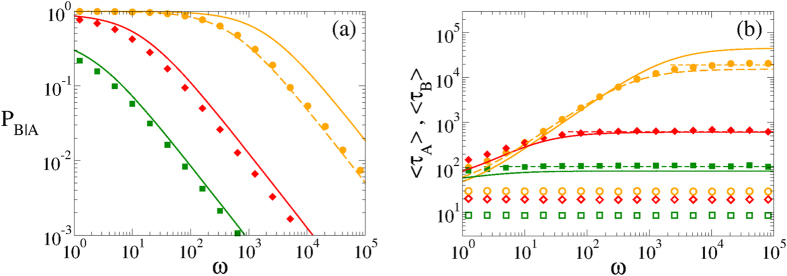
Statistics of outbreaks in a coupled two-layer system. Each layer is an ER network with mean degree 〈*k*〉 = 10. The coupling is *q* = 0.005 and the infection rates are *λ* = 0.115. Symbols represent MC simulation results for different system sizes: *N* = 250 (squares), *N* = 500 (diamonds) and *N* = 1000 (circles). (**a**) Outbreak probability in layer A after an outbreak in layer B, *P*_*B*|*A*_, vs the rewiring rate *ω*. Solid lines are the analytic approximations from Eq. (22), while the dashed line is the approximation Eq. (25) using the values *μ* = 6.49 × 10^−5^ and *β* = 0.0328/(1 + *ω*). (**b**) Mean outbreak times 〈*τ*_*A*_〉 (open symbols) and 〈*τ*_*B*_〉 (filled symbols) in networks A and B, respectively, vs *ω*. Solid curves are the analytic approximations from Eq. (23), while the dashed curve is the approximation Eq. (26). Horizontal dashed lines correspond to numerical results of the mean extinction times in network A.

In Fig. 6(a) we plot the probability of an outbreak in network B after an outbreak happened in network A, *P*_*B*|*A*_, as a function of the rewiring rate *ω*, obtained from MC simulations for various system sizes (solid symbols). We observe that *P*_*B*|*A*_ slowly decreases with *ω*, from a value *P*_*B*|*A*_(*ω* = 0) that approaches 1 as *N* increases (*P*_*B*|*A*_(*ω* = 0) = 0.881 and 0.998 for *N* = 500 and 1000, respectively). That is, for a static or very slowly varying internetwork connectivity, once the disease spreads on A then it almost always spreads on B but, as the speed of adaptation increases, the spreading likelihood is drastically reduced. For instance, for *ω* > 5 × 10^4^ and *N* = 1000, the outbreak in B is very unlikely (smaller than 1%), meaning that in most realizations the spreading on B does not happen. Thus, rewiring in finite networks proves to be very efficient in preventing the transmissibility between networks.

**Figure 7:**
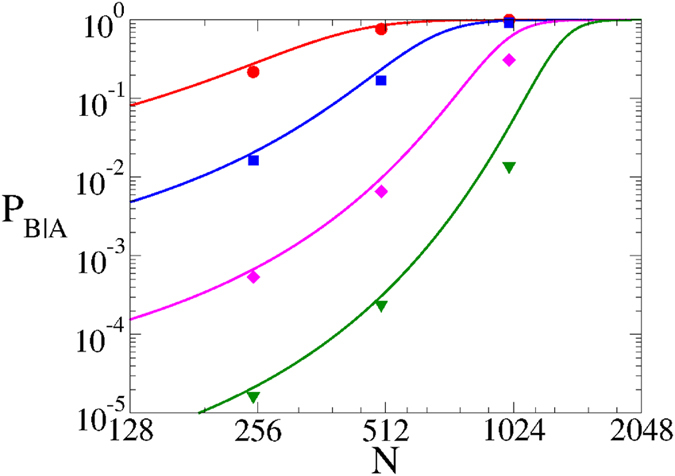
Outbreak probability in network B vs system size *N*. Parameter values are the same as in Fig. 6, with four different rewiring rates *ω* = 1.25 (circles), *ω* = 40 (squares), *ω* = 1280 (diamonds) and *ω* = 40960 (down triangles). Solid curves are the analytic approximations from Eq. (22).

In section “Methods” we develop an approach, based on a simple 3-state model, specially designed to account for the stochastic dynamics of the coupled system of two networks. This approach allows to obtain an approximate analytic expression for the outbreak probability. It reads


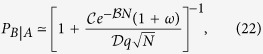


where the coefficients 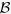, 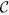 and 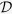 depend on *λ* and 〈*k*〉, and are given by Eqs (S12), (S13) and (S23), respectively, of the Supplementary Information. Solid curves in Fig. 6(a) correspond to Eq. (22). Even though we observe a discrepancy with the numerical values (symbols) that increases with the system size, Eq. (22) correctly captures the qualitative behavior of *P*_*B*|*A*_ with the model’s parameters. Discrepancies arise because *P*_*B*|*A*_ depends on the extinction rate *μ* (see Equation 25) calculated in section III of the Supplementary Information, which is underestimated by the analytical expression Eq. (S11). We observe from Eq. (22) that in a static network (*ω* = 0) *P*_*B*|*A*_ is close to 1 for large *N*. Also, *P*_*B*|*A*_ decays as 1/*ω* for large values of *ω*, and so it becomes zero only in finite networks with infinite large rewiring (*ω* → ∞). In the limit of infinite networks *N* → ∞, *P*_*B*|*A*_ → 1 for all finite *ω*, as we can observe in Fig. 7. This is in agreement with the mentioned fact that for infinite large systems in the strong endemic state, an outbreak in A always implies an outbreak in B. Therefore, we conclude that the rewiring has no effect on the outbreak probability for infinite networks, but as long as the networks are finite, its effect is amplified as *N* decreases.

In Fig. 6(b) we show mean outbreak times 〈*τ*_*A*_〉 and 〈*τ*_*B*_〉 in networks A and B, respectively, as a function of *ω*. The mean time 〈*τ*_*A*_〉 (〈*τ*_*B*_〉) is an average only over realizations in which network A (network B) undergoes an outbreak. We observe that 〈*τ*_*A*_〉 is very short and independent on *ω*, while 〈*τ*_*B*_〉 increases monotonically with *ω* and reaches a constant value as *ω* → ∞. The saturation level 〈*T*_*A*_〉 corresponds to the mean extinction time in network A when isolated (horizontal dashed lines). This result is a simple consequence of the fact that the outbreak in B always happens before the extinction in A. In section “Methods” we find the following approximate expression for the mean outbreak probability


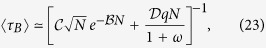


corresponding to the solid curves of Fig. 6(b). As it happens with *P*_*B*|*A*_, Eq. (23) captures the right qualitative behavior of 〈*τ*_*B*_〉, even though discrepancies with numerical values (symbols) increase with *N*. From Eq. (23) and Fig. 6(b) we see that for *ω* → 0 mean outbreak times in both networks are similar. In the opposite limit *ω* → ∞, 〈*τ*_*B*_〉 approaches the expression for the mean extinction time in network A (see section III of the Supplementary Information)


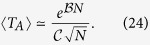


Even though the above analysis was done for a symmetric system, the analytical approached developed in section “Methods” can also be applied for a system with dynamical and structural asymmetries, i e., for *λ*_*A*_ ≠ *λ*_*B*_, 〈*k*_*A*_〉 ≠ 〈*k*_*B*_〉 and *N*_*A*_ ≠ *N*_*B*_. We can check that large systems, large infection rates and large connectivities favor the spreading and prevalence of the epidemics inside a network. Therefore, larger values of *λ*_*B*_, 〈*k*_*B*_〉 and *N*_*B*_ translate into a larger outbreak probability *P*_*B*|*A*_ and a smaller outbreak time 〈*τ*_*B*_〉. Also, by increasing *λ*_*A*_, 〈*k*_*A*_〉 and *N*_*A*_ the extinction in network A is delayed, thus increasing *P*_*B*|*A*_ and 〈*τ*_*B*_〉.

## Discussion

We studied the SIS model for disease spreading on two layers of networks coupled through dynamic interconnections. Links connecting infected and susceptible nodes in different layers are rewired at rate *ω*, thus the internetwork topology coevolves with the dynamics of disease spreading between both networks. We developed an analytical approach based on the pair approximation to explore the system’s dynamics. This approach revealed that the effect of the rewiring is to reduce the effective coupling for the transmission of the disease between the two layers, even though the number of interconnections is not affected by *ω*. We found two different mechanisms by which the rewiring can stop the propagation of the disease, effectively rescuing endemic states of the global interconnected system of two networks. Each of these mechanisms is efficient in one of the two considered scenarios, of weak and strong endemic states.

In weak endemic states, where the infection rate is below the epidemic threshold of a single layer, the two-layer system with dynamic interconnections can be thought as a system with static interconnections, but with an effective coupling that monotonically decreases with *ω*. Then, a sufficiently fast rewiring can reduce to zero the disease prevalence observed with static interconnections, by decreasing the intensity of the coupling to a very low level. This is described in statistical terms by means of a transition from an endemic to a healthy phase as *ω* overcomes a given threshold, which increases with the coupling and the networks’ connectivity.

In strong endemic states, where the infection rate is above the epidemic threshold of a single layer, the prevalence is largely reduced by rewiring, but it can only become zero for infinitely fast rewiring. The intuitive idea behind this result is that rewiring reduces the number of interlinks connecting infected and susceptible nodes between the networks. Then, when the rewiring is sufficiently large there are no *IS* links at the interface. As the disease is only transmitted through infected nodes, there is no possible spreading between networks, which behave as independent on the spreading on its partner network. Thus, layers are completely decoupled for the disease transmission.

The above results correspond to networks of infinite size. In finite systems, fluctuations play an important role in the final outcome. We run MC simulations to explore the spreading dynamics from an initial seed of infected nodes in one layer, and studied whether the disease was able to reach and spread in the other layer. We found that, in finite networks, there is a probability that the infection dies out in the initially infected layer before propagating to the other layer. This probability is practically negligible for a static interconnection, where the finite size of the network plays almost no role. However, interlink rewiring amplifies finite size effects, leading to a finite probability that the epidemics does not propagate to the second layer. The rewiring induces a delay in the outbreak of the susceptible layer that increases with *ω*, thus the outbreak may remain confined in the infected layer for arbitrary long times. The complementary probability that the disease spreads from the infected layer to the susceptible layer decreases monotonically with *ω*, and approaches zero as *ω* → ∞.

All the above results were checked by running Monte Carlo simulations on Erdös-Renyi networks with the same mean degree, but we expect them to be qualitatively the same on small world or scale-free networks, and also if both networks have different mean degrees (see section V of the Supplementary Information).

In summary, our results show that the adaptive rewiring of interlayer links is an effective strategy to control spreading across communities of individuals, either by the existence of a critical rewiring rate that effectively decouples the two communities, or by the amplification of finite size effects that prevent the propagation of the disease from one community to the other.

## Methods

### System setup and dynamics

To build the topology of the system we started from two Erdös-Renyi networks A and B, with the same number of nodes *N*_*A*_ = *N*_*B*_ = *N* and average degrees 〈*k*_*A*_〉 = 〈*k*_*B*_〉 = 〈*k*〉. Then, these networks were interconnected with links placed at random between nodes in A and B. We run the SIS dynamics on both networks, where infection, recovery and rewiring processes happen at rates *λ*, *μ* = 1 and *ω*, respectively. In the continuous time version of the model, at every infinitesimal time step *dt*, processes happen with probabilities *λdt*, *dt* and *ωdt*. To reduce computation times we implemented the Gillespie method^32^ to simulate the dynamics. At every iteration step of discrete time length Δ*t* = 1/*R*, one of the three processes is performed with respective probabilities *λ*/*R*, 1/*R* and *ω*/*R*, where *R* = *λ* + 1 + *ω* is the total rate.

### Critical rewiring

To calculate the critical rewiring rates of Fig. 2(b) we run “spreading experiments” in which, initially, one random node and their neighbors in each layer are infected (seeds). Depending on the parameter values, the seeds may grow and spread over the entire system. At the critical point, the probability that the system did not reach the healthy state up to time *t* decays as *P*(*t*) ~ *t*^−1^. We used two Erdös-Renyi networks of *N* = 10^6^ nodes and average degree 〈*k*〉 = 10 each, coupling *q* = 1.0 and infection rates *λ* = 0.0666, 0.07, 0.08, 0.09 and 0.095. The survival probability *P*(*t*) was calculated as the fraction of realizations that did not die up to time *t*, over a total of 50,000 independent realizations.

### Outbreak probabilities and times

We ran MC simulations of the dynamics on two coupled ER networks with *N* nodes each, mean degrees 〈*k*〉 = 10, coupling *q* = 0.005 and infection rates *λ*_*A*_ = *λ*_*AB*_ = *λ*_*B*_ = *λ*_*BA*_ = *λ* = 0.115. Because *λ* is above the critical value *λ*_*c*_ = 0.1, typically an initial seed in network A spreads over a large fraction of the system. When an outbreak happens in A, then the disease can pass to network B through interlinks, and may cause an outbreak in B as well. But, as we mentioned in the section for finite systems, this is not always the case. To quantify the outbreak, we estimated the probability *P*_*B*|*A*_ of an outbreak in network B given that there was an outbreak in network A, and the mean outbreak times 〈*τ*_*A*_〉 and 〈*τ*_*B*_〉. The conditional probability *P*_*B*|*A*_ measures the fraction of times that the rewiring is not able to stop the “transmission” of an outbreak from A to B. Outbreak times in A were calculated using a seed in A as initial condition. To calculate the outbreak statistics in B conditioned to outbreaks in A, we started the runs from a situation where there was already an outbreak in A, by randomly infecting a fraction *I*_*A*_ = 0.12 of nodes in A, above the prevalence level *I*^*s*^ = 0.11. Starting from a seed in A is not computationally efficient because there is a chance that the disease dies out in A before an outbreak occurs and, therefore, an outbreak in B neither takes place.

### Three-state symbolic model

In order to gain an insight about the statistics of outbreak times and their probabilities we need to consider the fluctuations associated with the system size. One way to incorporate the stochastic dynamics is to derive a system of equations as the one in section “Results”, but with additional noise terms corresponding to finite-size fluctuations. This procedure is in general very tedious and hard to carry out because of the large number of processes and equations involved and, besides, only numerical solutions can be obtained. That is why we develop here a much simpler approach, based on a synthetic model specially useful to investigate the outbreak probabilities and times. The approach consists on viewing the system and its dynamics at a coarse-grained level. We represent each network as a simple unit (node), and the state of each network as a binary variable, which is a symbolic representation of the two possible prevalence levels: *outbreak* when the density of infected nodes *I* fluctuates around 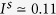 (active state), and *no-outbreak* when *I* is zero or close to zero (inactive state) [see Fig. 5]. This approach is an oversimplification of the complex dynamics of the system, but is able to reproduce rather well the collective properties of extinction and outbreak processes.

**Figure 8:**
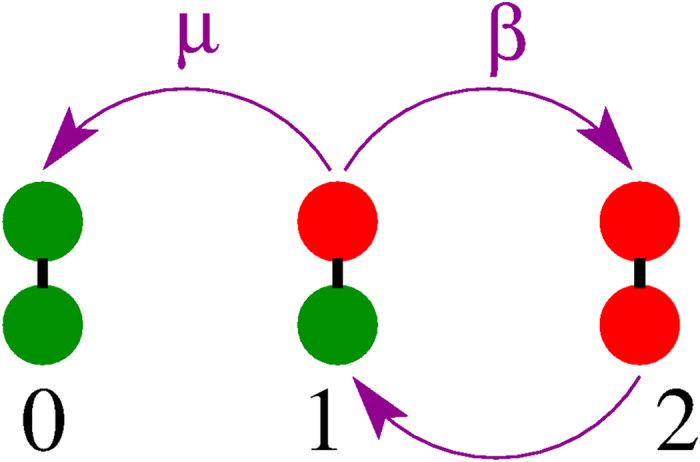
Schematic representation of the synthetic model. A red node represents an active network, while a green node denotes an inactive network. The transitions from one active network (state 1) to two inactive (state 0) and two active (state 2) networks happen at rates *μ* and *β*, respectively.

In Fig. 8 we show the possible states of the two-node system and their associated transitions. Red and green nodes represent, respectively, active and inactive networks. States 0, 1 and 2 denote the situations in which both networks are inactive, only one network is active, and both networks are active, respectively. These states represent the total activity of the system, so that the two cases A-active/B-inactive and A-inactive/B-active are grouped into a single state 1. An active node becomes inactive at rate *μ*, and activates its neighboring node at rate *β*. The dynamics of this synthetic model is analogous to the one of the SIS model, but with macroscopic transition rates *μ* and *β* that are *effective rates* that depend on the model’s parameters: the network size *N*, the infection rate *λ*, the mean connectivity 〈*k*〉, the interconnectivity *q*, and the rewiring rate *ω*. This model tries to capture the time evolution of the outbreak/extinction behavior of the coupled system of two networks. For instance, if at a given time network A is active and network B is inactive (state 1), we assume that network A triggers an outbreak in network B at constant rate *β* (1 → 2 transition), as in Fig. 5(a), and that the disease in network A dies out at constant rate *μ* (1 → 0 transition), as in Fig. 5(b).

Within this reduced model, the dynamics of transitions between states follows a multiple Poisson process with rates *μ* and *β*. Therefore, the outbreak probability in network B given an outbreak in A can be estimated as


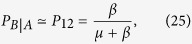


and the mean outbreak time in network B as


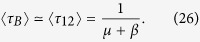


Here 〈*τ*_12_〉 is the mean time of the 1 → 2 transition normalized by *P*_12_, that is, over those realizations that made the transition to the outbreak. To check the validity of Eqs (25) and (26) we run MC simulations to calculate the rates *μ* and *β* for networks of size *N* = 10^3^ (see sections III and IV of the Supplementary Information). Dashed lines in Fig. 6(a,b) correspond to Eqs (25) and (26) using the numerical values of *μ* = 6.49 × 10^−5^ and *β* = 0.0328/(1 + *ω*). We observe that the agreement is good, showing that the symbolic model describes quite well the stochastic macroscopic dynamics of the system.

In sections III and IV of the Supplementary Information we also develop an analytical approach to obtain approximate expressions for *μ* [Eq. (S11)] and *β* [Eq. (S22)]. Plugging Eqs (S11) and (S22) into Eqs (25) and (26) we obtain the expressions for the outbreak in *B*, quoted in Eqs (22) and (23).

## Additional Information

**How to cite this article**: Vazquez, F. *et al*. Rescue of endemic states in interconnected networks with adaptive coupling. *Sci. Rep.* **6**, 29342; doi: 10.1038/srep29342 (2016).

## Supplementary Material

Supplementary Information
