# Supplementary material for: Rescue of endemic states in interconnected networks with adaptive coupling
Source: Sci Rep. 2016 Jul 6;6:29342. doi: 10.1038/srep29342 (PMC4933945; doi:10.1038/srep29342)
Supplement: Supplementary Information [file srep29342-s1.pdf]

SUPPLEMENTARY INFORMATION FOR:

Rescue of endemic states in interconnected networks with adaptive coupling

F. Vazquez,[1] M. Ángeles Serrano,[2,3] and M. San Miguel[4]

[1]*IFLYSIB, Instituto de Física de Líquidos y Sistemas Biológicos (UNLP-CONICET), 1900 La Plata, Argentina*
[2]*Departament de Física Fonamental, Universitat de Barcelona, Martí i Franquès 1, 08028, Barcelona, Spain*
[3]*Institució Catalana de Recerca i Estudis Avançats (ICREA), Barcelona 08010, Spain*
[4]*IFISC, Instituto de Física Interdisciplinar y Sistemas Complejos (CSIC-UIB), E-07122 Palma de Mallorca, Spain*

I. PAIR APPROXIMATION APPROACH

The time evolution of the model can be approximately described by the following set of ODEs for nodes and links, which account for state correlations between nearest-neighbors in the networks (first-order correlations):

$$\frac{dI_A}{dt} = -I_A + \lambda_A I_A S_A + \lambda_{BA} S_A I_B, \tag{S1a}$$

$$\frac{dI_A S_A}{dt} = 2I_A I_A - (1+\lambda_A) I_A S_A - 2\lambda_A I_A S_A I_A - \lambda_{BA} I_A S_A I_B + \lambda_A S_A S_A I_A + \lambda_{BA} S_A S_A I_B, \tag{S1b}$$

$$\frac{dI_A I_A}{dt} = -2I_A I_A + \lambda_A I_A S_A + 2\lambda_A I_A S_A I_A + \lambda_{BA} I_A S_A I_B, \tag{S1c}$$

$$\frac{dI_A S_B}{dt} = I_A I_B - (1+\omega) I_A S_B - \lambda_{AB} I_A S_B + \lambda_A I_A S_A S_B - \lambda_B I_A S_B I_B - 2\lambda_{AB} I_A S_B I_A + \lambda_{BA} I_B S_A S_B, \tag{S1d}$$

$$\frac{dS_A I_B}{dt} = I_A I_B - (1+\omega) S_A I_B - \lambda_{BA} S_A I_B + \lambda_B S_A S_B I_B - \lambda_A I_A S_A I_B - 2\lambda_{BA} I_B S_A I_B + \lambda_{AB} S_A S_B I_A, \tag{S1e}$$

$$\frac{dI_A I_B}{dt} = -2I_A I_B + \lambda_{AB} I_A S_B + \lambda_{BA} S_A I_B + \lambda_A I_A S_A I_B + \lambda_B I_A S_B I_B + 2\lambda_{AB} I_A S_B I_A + 2\lambda_{BA} I_B S_A I_B. \tag{S1f}$$

We have omitted here the rate equations for I_B, $I_B S_B$ and $I_B I_B$ because they have the same form as the equations for I_A, $I_A S_A$ and $I_A I_A$, respectively, after interchanging sub-indices A and B. Also, densities S_A, S_B, $S_A S_A$, $S_B S_B$ and $S_A S_B$ can be obtained from the conservation relations Eq. (1) of the main text. We note that the system of equations (S1) expresses the evolution of the densities that contain at least one infected node. This is particularly convenient when we analyze the stability of the healthy phase in section 2.2 of the main text.

As we can see, the rate equations for pairs depend on the densities of triplets, for which we use a notation analogous to the one used for nodes and links. For instance, $I_A S_B I_A$ represents the density per node of triples consisting of an infected node in A connected to a susceptible node in B that is in turn connected to another infected node in A. To close the system of equations (S1) we make use of the *pair approximation*, which assumes that the links of different types are homogeneously distributed over the networks. This allows to decompose triples in terms of nodes and pairs (see for instance [S1–S4]), closing the system at the level of pairs. The triplets of Eq. (S1) can be expressed as

$$\begin{aligned} I_A S_A I_A &= \frac{I_A S_A \cdot I_A S_A}{2S_A}\,;\;\; I_A S_A I_B = \frac{I_A S_A \cdot S_A I_B}{S_A}\,;\;\; I_A S_B I_A = \frac{I_A S_B \cdot I_A S_B}{2S_B}\,;\;\; I_A S_B I_B = \frac{I_A S_B \cdot I_B S_B}{S_B}, \\ I_A S_A S_A &= \frac{2 I_A S_A \cdot S_A S_A}{S_A}\,;\;\; I_A S_A S_B = \frac{I_A S_A \cdot S_A S_B}{S_A}\,;\;\; I_A S_B S_A = \frac{I_A S_B \cdot S_A S_B}{S_B}\,;\;\; I_A S_B S_B = \frac{2 I_A S_B \cdot S_B S_B}{S_B}. \end{aligned} \tag{S2}$$

Replacing the approximate expressions for the triplets from Eq. (S2) into the Eqs. (S1), we arrive to the system of Eqs. (2) of the main text.

II. PREVALENCE IN A SYMMETRIC TWO-LAYER SYSTEM

In this section we derive an analytic expression for the stationary density of infected nodes in a two-layer system with symmetric conditions. We start analyzing the case of a single isolated network, and then adapt the results for the case of two coupled networks.

Applying the pair approximation developed in section I for the case of a single network, for instance network A, we can describe the evolution of the system by the set of equations

$$\frac{dI_A}{dt} = -I_A + \lambda I_A S_A, \tag{S3a}$$

$$\frac{dI_A S_A}{dt} = 2I_A I_A - (1+\lambda) I_A S_A - \lambda \frac{(I_A S_A)^2}{S_A} + 2\lambda \frac{S_A S_A \cdot I_A S_A}{S_A}, \tag{S3b}$$

$$\frac{dI_A I_A}{dt} = -2I_A I_A + \lambda I_A S_A + \lambda \frac{(I_A S_A)^2}{S_A}, \tag{S3c}$$

together with the conservation relations

$$1 = I_A + S_A, \tag{S4a}$$

$$\frac{\langle k \rangle}{2} = S_A S_A + I_A I_A + I_A S_A, \tag{S4b}$$

where I_A, $I_A S_A$ and $I_A I_A$ are, respectively, the densities of I_A nodes, $I_A S_A$ links and $I_A I_A$ links. Notice that these equations can be derived from Eqs. (2) by setting $\lambda_{BA} = 0$ (A isolated from B). To find the stationary density of infected nodes I_A^s from Eqs. (S3), we set the derivatives to zero and express the stationary densities of links $[S_A S_A]^s$ and $[I_A S_A]^s$ and $[I_A I_A]^s$ in terms of I_A^s, using the relation Eq. (S4a). We obtain

$$[S_A S_A]^s = \frac{1 - I_A^s}{2\lambda}$$

$$[I_A S_A]^s = \frac{I_A^s}{\lambda}$$

$$[I_A I_A]^s = \frac{I_A^s}{2}\left[1 + \frac{I_A^s}{\lambda(1 - I_A^s)}\right].$$

Finally, plugging these expressions into Eq. (S4b) we arrive to the following quadratic equation for I_A^s

$$\lambda (I_A^s)^2 - \lambda(1 + \langle k \rangle) I_A^s + \lambda \langle k \rangle - 1 = 0. \tag{S5}$$

Only the solution of Eq. (S5) corresponding to the negative branch has a physical meaning, which reads

$$I_A^s = \frac{1 + \lambda_c}{2\lambda_c} - \sqrt{\left(\frac{1 - \lambda_c}{2\lambda_c}\right)^2 + \frac{1}{\lambda}}, \tag{S6}$$

where $\lambda_c = \langle k \rangle^{-1}$ is the critical infection rate of the network.

In the symmetric and homogeneous case scenario of two identical coupled networks A and B, with mean degree $\langle k \rangle$, infection rates $\lambda_A = \lambda_{AB} = \lambda_{BA} = \lambda_B = \lambda$ and coupling q, the static two-network system can be treated as a single network with mean degree $\langle k \rangle + \langle k_{AB} \rangle = (1 + q/2) \langle k \rangle$ and infection rate λ. Therefore, using Eq. (S6), the stationary density of infected nodes in the symmetric two-layer system is

$$I_{A,q}^s = \frac{1 + \lambda_{c,q}}{2\lambda_{c,q}} - \sqrt{\left(\frac{1 - \lambda_{c,q}}{2\lambda_{c,q}}\right)^2 + \frac{1}{\lambda}}, \tag{S7}$$

where $\lambda_{c,q} = [(1 + \frac{q}{2}) \langle k \rangle]^{-1}$ is the critical infection rate of the static coupled system. For a dynamic system with interlink rewiring ω, we showed in section 2.2 of the main text that the coupling becomes

$$q_\omega = \frac{q}{1 + \omega}, \tag{S8}$$

and thus we expect the stationary density of infected nodes to behave as

$$I_{A,q,\omega}^s = \frac{1 + \lambda_{c,q}^\omega}{2\lambda_{c,q}^\omega} - \sqrt{\left(\frac{1 - \lambda_{c,q}^\omega}{2\lambda_{c,q}^\omega}\right)^2 + \frac{1}{\lambda}}, \tag{S9}$$

with $\lambda_{c,q}^\omega = [(1+\frac{q_\omega}{2}) \langle k \rangle]^{-1}$ the infection threshold. Equation (S9) agrees reasonably well with the numerical integration of the system of Eqs. (2) for the two-network system (see Figs. 2 and 4(a) of the main text). The agreement is very good for ω close to $\omega_{c,q}^\lambda$, because the ansatz relation Eq. (S8) that we used for the effective coupling is only exact at the transition point (as we derived in section 2.2), that is, when $I_A = I_B = 0$. The agreement is also perfect for $\omega = 0$, because the solution Eq. (S7) is exact, while discrepancies arise between $\omega = 0$ and $\omega_{c,q}^\lambda$.

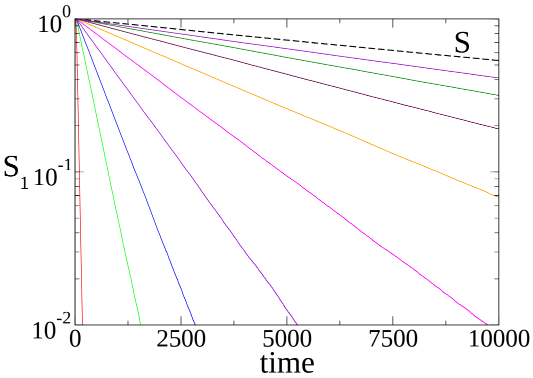

FIG. S1: **Time evolution of the persistence probability in state 1, S_1, for two coupled networks**. Each network has $N = 10^3$ nodes and mean degree $\langle k \rangle = 10$, the coupling is $q = 0.005$ and the infection rate is $\lambda = 1$. Each solid line corresponds to a different value of the rewiring rate $\omega = 1280, 640, 320, 160, 80, 40, 20, 10$ and 0 (from top to bottom). The dashed line at the top corresponds to the survival probability S of a single network. We note that the slope α of S_1 approaches to the slope μ of S as ω increases.

III. CALCULATION OF THE EXTINCTION RATE μ

To calculate the effective rate μ at which the epidemics extinguishes on a single isolated network, we run MC simulations on an ER network of $N = 10^3$ nodes, mean degree $\langle k \rangle = 10$ and infection rate $\lambda = 0.115$, starting from an initial density $I(0) = 0.11$ of infected nodes that corresponds to the stationary active state. Then, we calculate the survival probability $S(t)$, that is, the probability that the epidemics is not extinct up to time t. Working with survival probabilities rather than first-passage probabilities is better because fluctuations are smaller in the former case. Within the synthetic model, where we assume a constant decay rate from the active to the inactive state, we expect an exponential decay of the form $e^{-\mu t}$ for $S(t)$. In simulations, this decay is observed after a very short initial transient $\hat{t} \simeq 50$, during which S is nearly constant. Thus, we can approximate the shape of S as

$$S(t) \simeq \begin{cases} 1 & \text{for} \;\; t \le \hat{t} \\ e^{-\mu(t-\hat{t})} & \text{for} \;\; t > \hat{t}. \end{cases}$$

The slope of the S vs t curve on a linear-log plot gives $\mu \simeq 6.49 \times 10^{-5}$ (dashed line in Fig. S1).

An analytical expression for the upper bound of the average life time $\langle T \rangle$ of the SIS model on an arbitrary network was given by Van Mieghem in [S5]. It reads,

$$\langle T \rangle \simeq \frac{\frac{\lambda}{\lambda_c}\sqrt{2\pi}\, \exp\left\{ \left[\ln\left(\frac{\lambda}{\lambda_c}\right) + \frac{\lambda_c}{\lambda} - 1 \right] N \right\}}{\left(\frac{\lambda}{\lambda_c} - 1\right)^2 \sqrt{N}}, \tag{S10}$$

where N is the network size and λ_c is the epidemic threshold. Plugging the expression $\lambda_c = 1/\langle k \rangle$ for an ER network on Eq. (S10), and given that $\mu \simeq 1/\langle T \rangle$, we arrive to the following expression for a lower bound of the extinction rate

$$\mu \;\simeq\; \mathcal{C}\sqrt{N}\, e^{-\mathcal{B}N}, \tag{S11}$$

with

$$\mathcal{B} \;\equiv\; \ln\left(\lambda \langle k \rangle\right) + \frac{1}{\lambda \langle k \rangle} - 1 \quad \text{and} \tag{S12}$$

$$\mathcal{C} \;\equiv\; \frac{\left(\lambda \langle k \rangle - 1\right)^2}{\lambda \langle k \rangle \sqrt{2\pi}}. \tag{S13}$$

For $N = 10^3$, $\langle k \rangle = 10$ and $\lambda = 0.115$ we get $\mu \simeq 2.2 \times 10^{-5}$, which is approximately 1/3 of the numerical value $\mu \simeq 6.49 \times 10^{-5}$ obtained from simulations. The reason for this discrepancy is because we expect the numerical value of μ to be larger than the lower bound given by Eq. (S11). Even though Eq. (S11) is not precise, it captures the right qualitative behavior of μ with λ, $\langle k \rangle$ and N. Indeed, we notice that the coupling between the layers has no effect

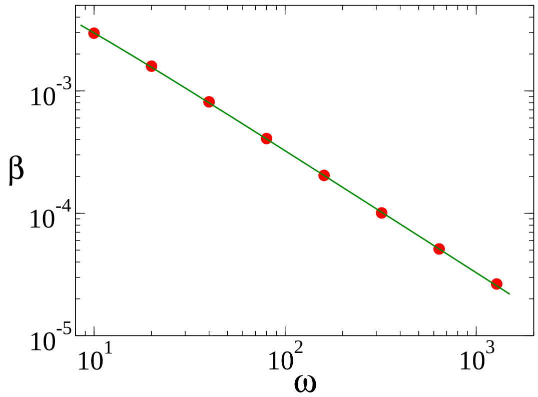

FIG. S2: **Outbreak transition rate** β **vs rewiring rate** ω. Solid circles were calculated from the slopes α of the persistent probability S_1 of Fig. S1, using the formula $\beta = \alpha - \mu$, with the extinction rate $\mu = 6.49 \times 10^{-5}$. The solid line corresponds to the fitting function $c/(1+\omega)$, where $c = 0.0328$ is the best fitting parameter.

on μ, as it is independent of q and ω. This is because we assume that the extinction in layer A is not affected by layer B, as long as B is in the inactive state (see Fig. 8 of the main text). We also observe that $\mu \to 0$ as $N \to \infty$, in agreement with the fact that for $\lambda > \lambda_c$ an infinite large system never decays to the healthy state. That is, once there is an outbreak in layer A, the transition back to the healthy state is only possible in finite systems.

IV. CALCULATION OF THE OUTBREAK RATE β

To obtain the outbreak rate β we run MC simulations on two interconnected ER networks A and B, of $N = 10^3$ nodes each, mean degree $\langle k \rangle = 10$, $\lambda = 0.115$ and coupling $q = 0.005$, starting from a density $I_A(0) = 0.11$ and $I_B(0) = 0$ of infected nodes in A and B, respectively. We stop the simulation when either I_A becomes zero ($1 \to 0$ transition) or I_B overcomes the value $I^s = 0.11$ by the first time ($1 \to 2$ transition). This corresponds to having the system initially in state 1, with A active and B inactive (see Fig. 8 of the main text), and calculating the first-passage statistics to either state 0 or 2. Given that β is a measure of the likelihood that the disease is transmitted from A to B, we expect β to decrease as the rewiring rate ω increases. Therefore, we run simulations for several values of ω to study this dependence. To obtain the transition rates, it proves useful to work with the persistence probability in state 1, $S_1(t)$, i e., the probability that the system did not leave state 1 up to time t. Given that the total outgoing rate from state 1 is $\mu + \beta$, we expect the persistence probability to behave as

$$S_1(t) \simeq e^{-(\mu+\beta)t}. \tag{S14}$$

As it happens for the single-network survival probability $S(t)$, we found from MC simulations that $S_1(t)$ is nearly constant up to a time $\hat{t} \simeq 50$, and then decays exponentially fast to zero (see Fig. S1):

$$S_1(t) \simeq \begin{cases} 1 & \text{for} \quad t \leq \hat{t} \\ e^{-\alpha(t-\hat{t})} & \text{for} \quad t > \hat{t}, \end{cases} \tag{S15}$$

where the exponent α depends on the rewiring rate ω. Again, it seems that after a time $\hat{t}$ the dynamics of the two-network system behaves, at a coarse-grained level, as the one of a three-state system with effective transition rates. We observe in Fig. S1 that S_1 approaches S as ω increases. This is consistent with the fact that at very large rewiring rates network A decouples from network B. Thus, A behaves as a single network, independent from B and, therefore, the only possible process is the extinction from the initial active state in A.

Comparing Eqs. (S14) and (S15) we obtain the relation

$$\alpha = \mu + \beta. \tag{S16}$$

In Fig. S2 we plot $\beta = \alpha - \mu$ vs ω, using the measured values of α from Fig. S1 and the value $\mu = 6.49 \times 10^{-5}$ calculated in section III. The data is well fitted by the function $\beta(w) = c/(1+\omega)$, with $c = 0.0328$. This means that β vanishes as ω goes to infinity, showing that the outbreak transition $1 \to 2$ is strictly zero only in the $\omega \to \infty$ limit.

We now obtain an analytical expression for β. Starting from an initial condition consisting of an outbreak in network A and no infected nodes in network B (state 1), we assume that a node in B gets infected ("infection seed") at a rate Λ, and that this seed grows generating an outbreak with probability S_∞. Therefore, β is estimated as

$$\beta = \Lambda\, S_\infty. \tag{S17}$$

That is, every mean time interval $1/\Lambda$ a seed is planted in B, which grows and becomes an outbreak with probability S_∞, and thus the outbreak probability per time is as in Eq. (S17). Here

$$S_\infty \equiv \lim_{t\to\infty} S(t),$$

where $S(t)$ is the survival probability in a spreading experiment, i e., the probability that a run starting from a seed survives up to time t. Based on a rigorous proof for spreading models with absorbing states [S6], we expect the ultimate survival probability to be equivalent to the density of nodes in the stationary state,

$$S_\infty = I^s. \tag{S18}$$

Besides, the total infection rate from A to B, Λ, should be proportional to the total number of interlinks of type $I_A S_B$ and the infection rate per link λ, that is

$$\Lambda = \lambda N I_A S_B. \tag{S19}$$

Here $I_A S_B$ is the stationary density of links connecting an infected node in A and a susceptible node in B, which is estimated as $I_A S_B = I_A^s \langle k_{AB}\rangle = I_A^s\, q \langle k\rangle /2(1+\omega)$, given that all nodes in B are susceptible. We have also used the effective coupling $q_\omega = q/(1+\omega)$ for a dynamic network, which accounts for the reduction of the number of interlayer links between infected and susceptible nodes when the rewiring is switched on. Plugging this expression for $I_A S_B$ into Eq. (S19) we obtain

$$\Lambda = \frac{\lambda\, q \langle k\rangle N I_A^s}{2(1+\omega)}. \tag{S20}$$

Finally, replacing the expressions for S_∞ and Λ from Eqs. (S18) and (S20) into Eq. (S17) leads to

$$\beta = \frac{\lambda\, q \langle k\rangle N (I_A^s)^2}{2(1+\omega)}. \tag{S21}$$

Using the values of the model's simulations $N = 10^3$, $\langle k\rangle = 10$, $\lambda = 0.115$ and $I_A^s = 0.11$ in Eq. (S21) we get $\beta = c/(1+\omega)$, with $c \simeq 0.0348$, which is comparable to the best fitting parameter 0.0328 of Fig. S2 (about 6% difference). This shows that Eq. (S21) is a good approximation for β, for the parameters used in the simulation.

To complete the analytic expression for β in terms of the model's parameters, we insert into Eq. (S21) the analytical expression Eq. (S6) for I_A^s obtained in section II, and replace back λ_c by $\langle k\rangle^{-1}$. We finally arrive to

$$\beta \simeq \frac{\mathcal{D}\, q\, N}{1+\omega}, \tag{S22}$$

with

$$\mathcal{D} \equiv \frac{\lambda \langle k\rangle}{2}\left(\frac{1+\langle k\rangle^2}{2} + \frac{1}{\lambda} - \frac{1+\langle k\rangle}{2}\sqrt{(1-\langle k\rangle)^2 + \frac{4}{\lambda}}\right). \tag{S23}$$

From Eq. (S22) we see that β increases linearly with q and N. This happens because β is proportional to the total number of infections from nodes in A to nodes in B through interlayer links, which are proportional to q and N. In particular, A has no effect on B ($\beta = 0$) when $q = 0$. We also observe that β decreases monotonically with ω, showing that the rewiring reduces the transmission rate of the disease from A to B.

V. ASYMMETRIC CASE $\langle k_A\rangle \neq \langle k_B\rangle$

In order to check that the endemic-healthy transition is also observed for an asymmetric system, we run Monte Carlo simulations for two interconnected networks of different mean degrees. We start from the static ($\omega = 0$) symmetric

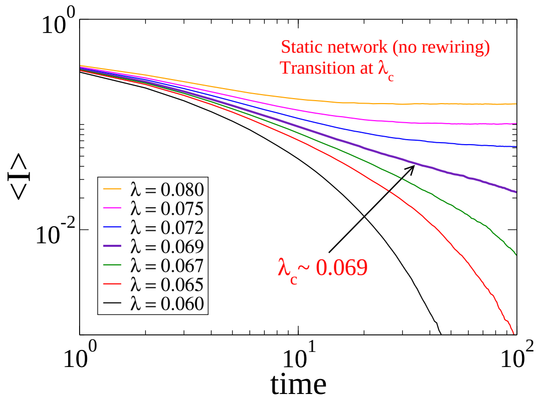

FIG. S3: **Time evolution of the average density of infected nodes $\langle I \rangle$ for two static and symmetric interconnected networks, and various values of the infection rate λ.** Networks have mean degrees $\langle k_A \rangle = \langle k_B \rangle = 10$, and $N = 10^3$ nodes each. The coupling is $q = 1.0$.

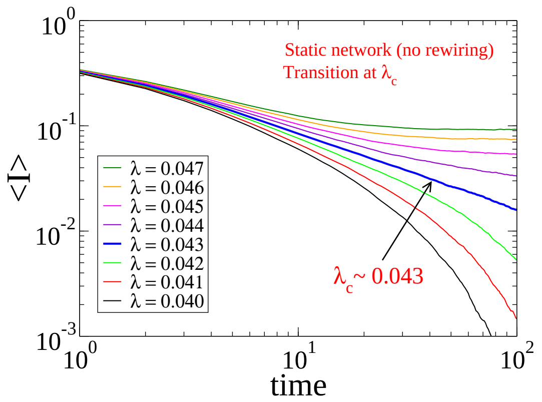

FIG. S4: **Time evolution of the average density of infected nodes $\langle I \rangle$ for two static and asymmetric interconnected networks, and various values of the infection rate λ.** Networks have mean degrees $\langle k_A \rangle = 10$ and $\langle k_B \rangle = 20$, and $N = 10^3$ nodes each. The coupling is $q = 1.0$.

case with $\langle k_A \rangle = \langle k_B \rangle = 10$ and coupling $q = 1.0$, where we find the critical infection rate $\lambda_c \simeq 0.069$ (see Fig. S3). At the transition point, the average density of infected nodes in the two-network system $\langle I \rangle$ decays to zero as a power law. In Fig. S4 we see that the transition decreases to $\lambda_c \simeq 0.043$ when we consider the asymmetric case of two static networks with mean degrees $\langle k_A \rangle = 10$ and $\langle k_B \rangle = 20$. We then set the system in the endemic phase by fixing $\lambda = 0.047 > 0.043 = \lambda_c$, and start increasing the rewiring ω (Fig. S5). We observe that when ω overcomes the threshold value $\omega_c \simeq 0.5$, $\langle I \rangle$ decays to zero, indicating the transition to the healthy phase.

[S1] M. J. Keeling, Proc. Roy. Soc. Lond. B **266**, 859 (1999).
[S2] T. Gross, C. J. D. D?Lima, and B. Blasius, Phys Rev Lett **96**, 208701 (2006).
[S3] S. Shai and S. Dobson, Phys Rev E **87**, 042812 (2013).
[S4] G. Demirel, F. Vazquez, G. Böhme, and T. Gross, Physica D **267**, 68 (2014).
[S5] P. Van Mieghem, arXiv:1310.3980 (2013).
[S6] R. Durrett, Lecture Notes on Particle Systems and Percolation (Wadsworth & Brooks/Cole, Pacific Grove, CA, 1988).

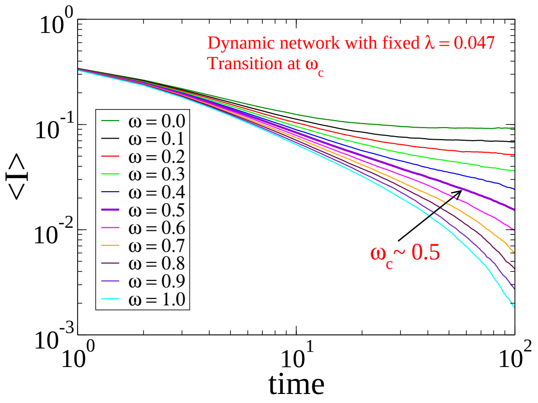

FIG. S5: **Time evolution of the average density of infected nodes $\langle I \rangle$ for two dynamic and asymmetric interconnected networks, and various values of the rewiring rate ω.** Networks have mean degrees $\langle k_A \rangle = 10$ and $\langle k_B \rangle = 20$, and $N = 10^3$ nodes each. The coupling is $q = 1.0$ and the infection rate is fixed to $\lambda = 0.047$, above the critical value in a static system $\lambda_c = 0.043$.
